# Stable Knockdown of Adenosine Kinase by Lentiviral Anti-*ADK* miR-shRNAs in Wharton’s Jelly Stem Cells

**DOI:** 10.22074/cellj.2018.4916

**Published:** 2018-01-01

**Authors:** Hajar Estiri, Ali Fallah, Masoud Soleimani, Abbas Aliaghaei, Fariba Karimzadeh, Shahnaz Babaei Abraki, Mohammad Hossein Ghahremani

**Affiliations:** 1 Department of Molecular Medicine, School of Advanced Technologies in Medicine, Tehran University of Medical Sciences, Tehran, Iran; 2 Iranian Institute of Cell and Gene therapy, Tehran, Iran; 3 Bioviva Science USA, Seattle, USA; 4 Department of Hematology, Faculty of Medical Sciences, Tarbiat Modares University, Tehran, Iran; 5 Neuroscience Lab, Department of Anatomy and Cell Biology, School of Medicine, Shahid Beheshti University of Medical Sciences, Tehran, Iran; 6 Cellular and Molecular Research Center, Iran University of Medical Sciences, Tehran, Iran; 7 Shefa Neuroscience Research Center, Khatam-al-Anbia Hospital, Tehran, Iran; 8 Department of Toxicology and Pharmacology, Faculty of Pharmacy, Tehran University of Medical Sciences, Tehran, Iran

**Keywords:** Adenosine Kinase, Gene Knockdown Techniques, Lentivirus, RNA Interference, Wharton’s Jelly

## Abstract

**Objective:**

In this study, we describe an efficient approach for stable knockdown of adenosine kinase (*ADK*) using lentiviral system, in an astrocytoma cell line and in human Wharton’s jelly mesenchymal stem cells (hWJMSCs). These sources of stem cells besides having multilineage differentiation potential and immunomodulatory activities, are easily available in unlimited numbers, do not raise ethical concerns and are attractive for gene manipulation and cell-based gene therapy.

**Materials and Methods:**

In this experimental study, we targeted adenosine kinase mRNA at 3' and performed coding sequences using eight miR-based expressing cassettes of anti-*ADK* short hairpin RNA (shRNAs). First, these cassettes with scrambled control sequences were cloned into expressing lentiviral pGIPZ vector. Quantitative real time-polymerase chain reaction (qRT-PCR) was used to screen multi-cassettes anti-*ADK* miR-shRNAs in stably transduced U-251 MG cell line and measuring *ADK* gene expression at mRNA level. Extracted WJMSCs were characterized using flow cytometry for expressing mesenchymal specific marker (CD44+) and lack of expression of hematopoietic lineage marker (CD45-). Then, the lentiviral vector that expressed the most efficient anti-*ADK* miR-shRNA, was employed to stably transduce WJMSCs.

**Results:**

Transfection of anti-*ADK* miR-shRNAs in HEK293T cells using CaPO_4_ method showed high efficiency. We successfully transduced U-251 cell line by recombinant lentiviruses and screened eight cassettes of anti-*ADK* miR- shRNAs in stably transduced U-251 MG cell line by qRT-PCR. RNAi-mediated down-regulation of *ADK* by lentiviral system indicated up to 95% down-regulation of *ADK*. Following lentiviral transduction of WJMSCs with anti-*ADK* miR- shRNA expression cassette, we also implicated, down-regulation of *ADK* up to 95% by qRT-PCR and confirmed it by western blot analysis at the protein level.

**Conclusion:**

Our findings indicate efficient usage of shRNA cassette for *ADK* knockdown. Engineered WJMSCs with genome editing methods like CRISPR/cas9 or more safe viral systems such as adeno-associated vectors (AAV) might be an attractive source in cell-based gene therapy and may have therapeutic potential for epilepsy.

## Introduction

Previous molecular studies indicated that up-regulation of adenosine kinase (*ADK*), a key enzyme in the metabolism of adenosine, is one of the most important processes involved in astrogliosis (1, 2). RNA interference (RNAi) or post-transcriptional gene silencing (PTGS) is an interesting molecular tool for gene knockdown. Knock down of *ADK* increases intracellular adenine and results in extracellular adenosine augmentation. Adenosine has known protective effects on the central nervous system (3, 4). *ADK* gene could be targeted by RNAi in human cells which is an effective way to produce adenosine-releasing cells (5, 6). Adenosine augmentation exhibits a paracrine therapeutic effect and has potential for therapeutic applications in neurological diseases like refractory epilepsy (7). 

Among children, the highest incidence of epilepsy is seen at ages less than five years old. Therefore, finding a new source of cells with therapeutic applications is highly required (8). Wharton’s jelly stem cells (WJMSCs) are an alternative for bone marrow mesenchymal stem cell (BMSCs). They are multipotent cells which are easily isolated in unlimited numbers with long-term ex vivo proliferation and immunomodulatory properties (9). WJMSCs are obtained from discarded human umbilical cord, with no ethical concern (10). These cells express specific MSCs markers like CD44 and are negative for CD45 hematopoietic lineage marker (11, 12). Being easily accessible, makes WJMSCs an alternative and attractable source for cell-based gene therapy.

In the present study, we used anti-*ADK* microRNA (miR) in a shRNA lentiviral systems (miR-shRNA) for ex vivo gene therapy in U-251 MG cell line. We screened eight cassettes of miR-shRNAs that target human *ADK* gene. In order to screen and select the most efficient anti-*ADK* miR-shRNA, astrocytoma cell line was employed. Human U-251 MG cell line highly expresses *ADK* gene. Pseudo lentiviruses of eight anti-*ADK* miR-shRNAs were used for transducing of astrocytoma cell lines. The most efficient anti-*ADK* miRshRNA for knockdown of *ADK* was selected by quantitative real time-polymerase chain reaction (qRT-PCR) analysis of established cells. Furthermore, human WJMSCs were isolated and cultured after characterizing with flow cytometry for specific mesenchymal markers. Knockdown of *ADK* gene in WJMSCs was confirmed by western blot analysis as well as qRT-PCR after transduction using the most efficient anti- *ADK* miR-shRNA lentiviral vector. 

## Materials and Methods

In this experimental study, human U-251 MG cell line (Sigma-Aldrich, USA) was cultured with Dulbecco’s Modified Eagle’s Medium (DMEM, Gibco-BRL, Japan) and 10% fetal bovine serum (FBS, Gibco, USA). This cell line highly expresses *ADK* gene. The third passage of these cells was used for screening the anti-*ADK* miR-shRNAs to knockdown *ADK* gene. All the experiments including animal works were approved by TUMS Ethics committee No. 9301- 87-25045-109011 and were performed based on the committee guideline. 

### Lentiviral constructs for the expression of anti-*ADK* miR-shRNAs

The eight different pre-miRNAsequences and a randomizedscrambled control (SC) sequence were purchased (GE Healthcare). All miR-shRNA cassettes were cloned into the pGIPZ lentiviral vector, which contained a TurboGFPgreen fluorescence protein (tGFP) as a reporter gene, internalribosome entry site (IRES) and a puromycin resistance gene; thus, it allowed co-expression of the respective miR-shRNAwith tGFP and selection of stably transduced cells withpuromycin. Expression of tGFP, puromycin and miR-shRNAs were under Cytomegalovirus (CMV) constant promoter. All genes were expressed as a single mRNA. At first, mRNA wasprocessed in nuclear for producing premature miR-shRNAand bicistronic GFP-puromycin mRNA. pGIPZ lentiviralexpression vector harbored internal long terminal repeats(LTRs) zeocin selection marker for selection of correct intact vector during bacterial propagation.

### Production of recombinant pseudo lentiviruses

Recombinant lentiviruses were produced according to the Prof. Trono lab protocol with some modifications (13, 14). Briefly for all 8 miR-shRNAs and the positive control vector, 1×10^6^ HEK 293T cells (Invitrogen, USA) were cultured in a 10-cm^2^ plate in DMEM medium supplemented with 10% FBS one day prior to transfection. Two hours before transfection, the medium was replaced with fresh medium. The transfection mixture contained 21 µg of pGIPZ-miR- sh/SC, 15 µg of pCMV-dR8.2, 10.5 µg of pMD2, 33 µl of TE 1X, 105 µl of 2.5 M CaCl_2_, and 1064 µl of 2X HEPES- buffered saline (HeBS) finally reaching a volume of 2100 µl using buffered water. 

All the components were mixed, and HeBS 2X was addedwhen the solution was being vortexed vigorously. The final volume of transfection mixture used for each 10-cm^2^ plate, was 2100 µl. HEK 293T cells were incubated withtransfection solution in 37°C for 14 hours. The transfection medium was replaced with fresh medium 14 hours aftertransfection and cells were assayed for GFP expression usinga fluorescent microscope (LaboMed, USA). GFP expressionindicated transfection efficiency. To estimate the transfectionrate, five fields were randomly observed under the fluorescentmicroscope. Supernatant of cells containing recombinantviruses was collected at three time points (24, 48 and 72 hoursafter transfection) after 14 hours post-transfection. Next, collected supernatants were centrifuged at 180 g and filteredthrough a 0.25 µm filter before concentration and titration. For concentration of the supernatants, we used PEG 6000 and9000 g centrifuge. The lentiviral titers from the 9 different miR-shRNA constructs due to tGFP gene in our vectors weredetermined using flow cytometry method. The determinationof the lentiviral titer allowed us to estimate the multiplicity ofinfection (MOI) and thus to reduce the infectious activity of the viral stocks.

### Determination of lentivirus titration

Since the stocks of vector carry GFP transgene that can beeasily monitored by flow cytometer, we used this method fortitration of lentivectors. We used Prof. Trono lab protocol forlentivirus titration (15). For this purpose, 293T cells werecultured in DMEM medium supplemented with 10% FBS in a12-well plate with 1×10^5^ cells in each well. Then, the medium was removed and cells were transduced in 500 µl of fresh DMEM with serial dilutions of the vector that correspondedto the final amount of 1, 10^-1^, 10^-2^, 10^-3^, 10^-4^ and 10^-5^ µl of the vector. After 24 hours, the medium was removed and one ml of fresh medium was added to each well. Then, 72 hours after transduction, cells were processed for Fluorescence ActivatedCell Sorting (FACS) analysis. The following formula wasused for calculating titer by flow cytometry:

"Titer HEK 293T transducing U/ml=[Number of target cells(counted on day 1)×(% of GFP-positive cells/100)]/volume of supernatant (ml)"

Ideally, the cells should present 2-20% GFP^+^ to confirm that cells that received multiple copies of the virus, were not counted. The flow cytometer apparatus did not have enough sensitivity for determination of GFP^+^ <1%.

### Genetic engineering of U-251 MG cell line

After three passages, U-251 MG was cultured in six-wellcell culture plates at a density of 2×10^5^ cell /well /2 ml. Beforeadding fresh recombinant viruses, U-251 MG cell line waswashed with PBS. Recombinant viruses in culture medium with an MOI of 5 were used for transduction of cells in each well. To increase the rate of transduction, the spinfectionmethod was used. After infection, the plates were incubated at 37°C and the medium was changed 24 hours later. Transducedcells were assayed for GFP expression with a fluorescentmicroscope (LaboMed, USA), 72 hours after transduction. GFP expression indirectly indicated the expression of miRshRNA and transduction efficiency. To select stably transducedcells, from day 4, the medium was replaced with fresh mediumcontaining 2 µg/ml puromycin for 5-7 days.

### Quantitative analysis of *ADK* knockdown by real-time analysis in U-251 MG cells

A QuantiFast SYBR Green RT-PCR Kit (Qiagen, USA) was employed to determine and monitor *ADK* expression afterknockdown of this gene by recombinant lentiviral constructsthat express anti-*ADK* miR-shRNAs. After puromycinselection, 2×10^6^ cells from stable U-251 MG cell line was used for RNA extraction and cDNA synthesis (Qiagen kit, USA). According to the manufacturer protocols, cDNA wasused for standard real-time PCR. Specific primer pairs wereused for *ADK* and *TBP* (TATA Sequence-Binding Protein). The expression of ADK mRNA was evaluated in the lentiviralengineered U-251 MG cells. For improving the reliability ofrelative RT-PCR, *TBP* was used as a reference gene.

### Isolation and expansion of human Wharton’s jelly mesenchymal stem cells 

Human Wharton’s jelly tissues were obtained from newborn umbilical cord in accordance with bioethics agreement following obtaining the consent from its mother at Taleghani Hospital, Tehran, Iran. Mucoid connective tissueor Wharton’s jelly were separated from blood vessels (twoarteries and one vein of the umbilical cord) then, washedtwice with PBS containing penicillin, streptomycin andamphotericin. The cord was rinsed with PBS and isolatedfrom amniotic membrane. After separation of the matrixfrom cord vessels, the jelly matrix was cut into small piecesand transferred into culture dishes supplemented with DME/ F12 medium, 10% FBS and antibiotics. Two weeks later, the tissues were discarded and the isolated growing cells(WJMSCs) were fed with the same medium. The cells weregrown to 60% confluence and passaged by trypsinization. The third passage of WJMSCs was used for characterization.

### Flow cytometry

A number of 1×10^5^ cultured WJMSCs were washed, fixed and incubated for 15 minutes at 4°C with a 1:9 dilution of normal goat serum in phosphate buffered saline (PBS, Sigma- Aldrich, USA). Then, cells were incubated for 1 hour withFITC-conjugated antibodies (CD44 and CD45) for labelling. Cells washed with 2% FBS in PBS were used for analyzingwith FACSCalibur apparatus (Becton Dickenson, USA). Thecontrol population was stained with isotype-matched antibodies(FITC-conjugated and PE-conjugated mouse IgG monoclonalisotype standards) and confirmed by positive fluorescence ofthe limbal samples. For each sample, at least 1×10^4^ events were recorded and analyzed by WinMDI software (USA).

### Quantitative analysis of *ADK* knockdown by selected anti-*ADK* miR-shRNA in Wharton’s jelly mesenchymal stem cells

A QuantiFast SYBR Green RT-PCR Kit (Qiagen, Germany) was employed to monitor knockdown of *ADK* after transduction of WJMSCs by the most efficient recombinant lentiviral construct that expressed anti-*ADK* miR-shRNA. After puromycin selection with 2 µg/ml for 5-7 days, 1×10^6^ cells from WJMSCs were used for RNAextraction and cDNA synthesis (Qiagen, Gemany). In the second step, cDNA was used for standard real-time PCR. Specific primer pairs were used for *ADK* and *TBP*. The expression of *ADK* mRNA was evaluated in the lentiviral-engineered WJMSCs cells.

### Preparation of total protein extracts

Stable transduced umbilical cord mesenchymal stem cells (WJMSCs) were lysed using ReadyPrep™ mammalian cell lysis reagent (Bio-Rad) comprising complete protease inhibitor cocktail. Cellular proteins were prepared according to manufacturer’s protein extraction protocol. The samples were stored at -80°C until western blot analysis. The protein content of sample lysate was measured using Bradford’s method (16). 

### Western blot analysis

An equal amount of proteins was loaded on 12% sodium dodecyl sulfate-polyacrylamide gel electrophoresis (SDSPAGE) and transferred to polyvinylidene fluoride membranes. Blots were blocked using non-fat dry milk for 1 hour. After blocking, membranes were probed with *ADK* primary antibody (Santa Cruz, 1/1000). Then anti-rabbit IgG antibody conjugated with horseradish peroxidase (Santa Cruz, 1/8000) was used for 1 hour at room temperature. Subsequently, the blots were reprobed with a ß-actin antibody (1:2000, Abcam, ab8227, USA). Then, the blots were treated with Electro Chemi Luminescence reagents (Amersham Biosciences, UK). For quantification of protein intensities, western blot bands were visualized on radiograph film and evaluated by Image J software. Human umbilical cord tissue was used in accordance with the Declarations of Tehran University of Medical Science and Stem Cell Research Center Committee after obtaining written informed consent from the mother.

## Results

### Transfection efficiency of lentiviral anti-*ADK* miRshRNAs vectors in packaging 293T cell line

**Fig.1:**
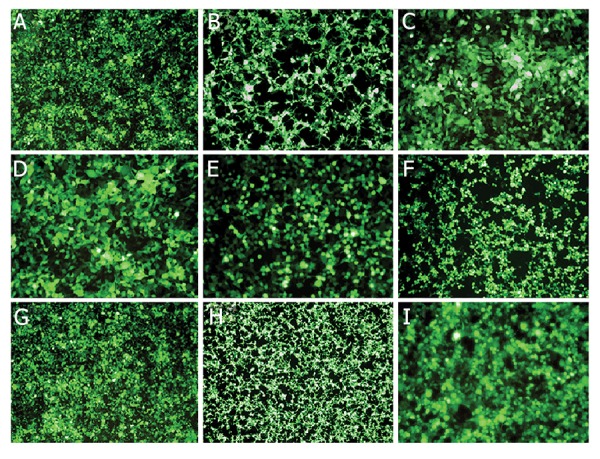
Transfection efficiency of lentiviral anti-*ADK* miR-shRNAs vectors in packaging 293T cell line. A-H. HEK 293T cells were transfected with anti-*ADK* miRshRNAs and I. Scrambled control and with high efficiency (90-95%). The rate of GFP expression evaluated undera fluorescent, microscope confirmed the high efficiency of transfection and expression of anti-*ADK* miR-shRNAs.

All of nine transfer vectors (eight anti-*ADK* miRshRNAs and scrambled control) with helper vectors (pCMV-dR8.2 and pMD2) were separately co-transfected into HEK293T cell line using CaCl_2_ protocol. The transfection efficiency, as evaluated based on GFP marker under the fluorescent microscope, was more than 90-95% for eight anti-*ADK* miR-shRNAs (Fig .1) and scrambled control.

### Titration of lentivirus by FACS

The titer of viral particles was approximately 1.5-2×10^7^ U/ml. Serial dilutions of the vector that corresponded to the final amount of 10^-3^-10^-4^ µl of the vector, were used for calculating the titration because the GFP^+^ cells in other serial dilutions were more than 20% or under 1%.

### Transduction of U-251 MG cell line

**Fig.2:**
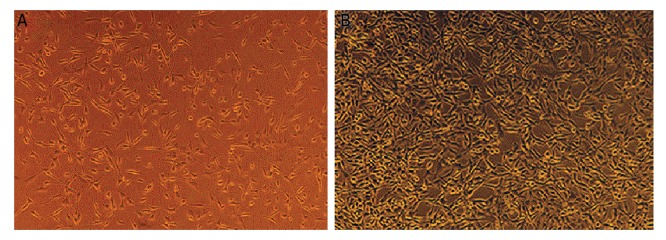
Morphological characteristics of U-251 MG cells. A. These cells are shown under a phase-contrast microscope with low confluence and B. High confluence.

**Fig.3:**
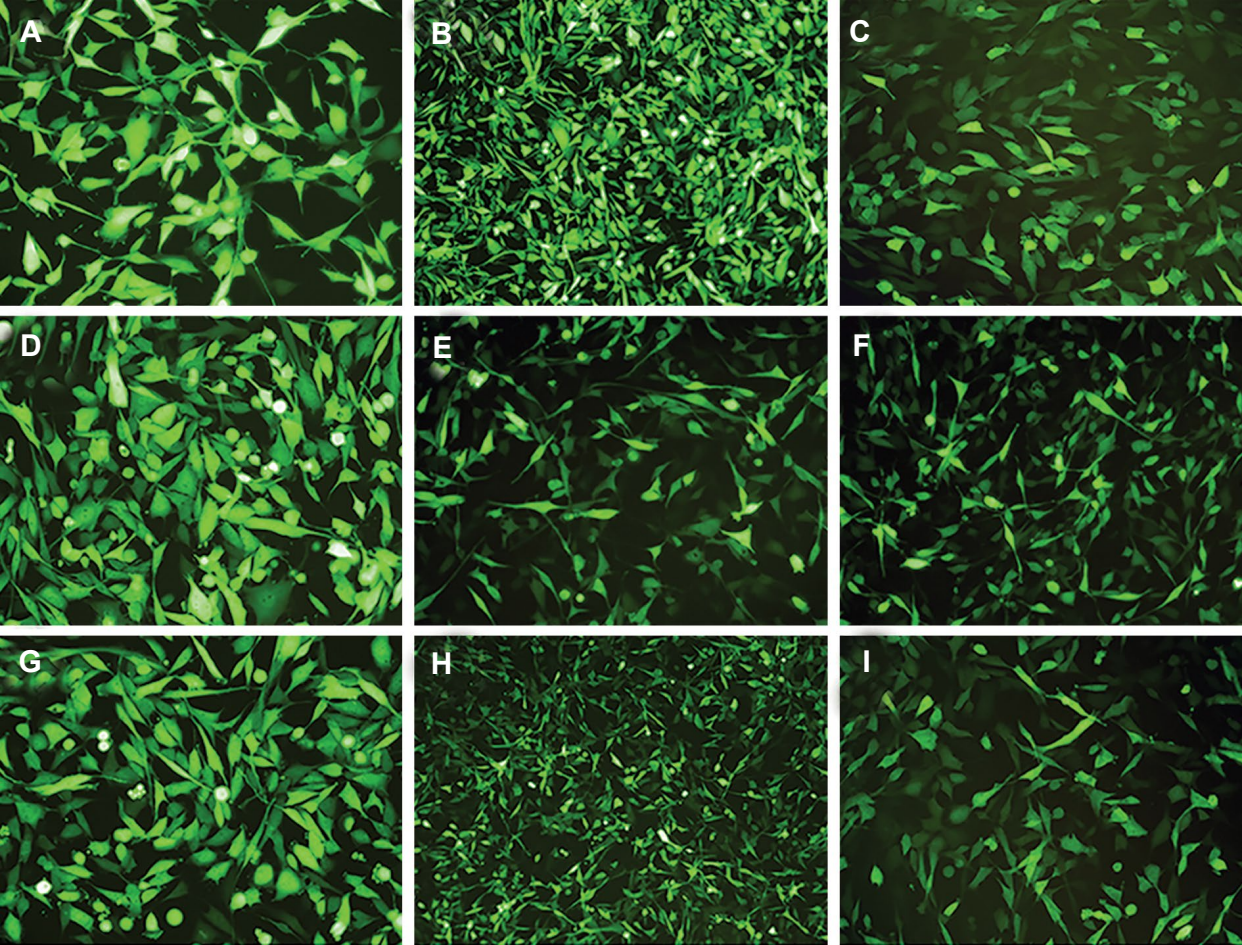
Lentiviral Transduction efficiency in U-251 MG cell line. High rate of tGFP expression was seen under a fluorescent microscope 72 hours after transduction with anti-*ADK* miR-shRNAs lentiviral vectors. A-H. Transduction with sh1-sh8 and I. Transduction of cells with scrambled control.

**Fig.4:**
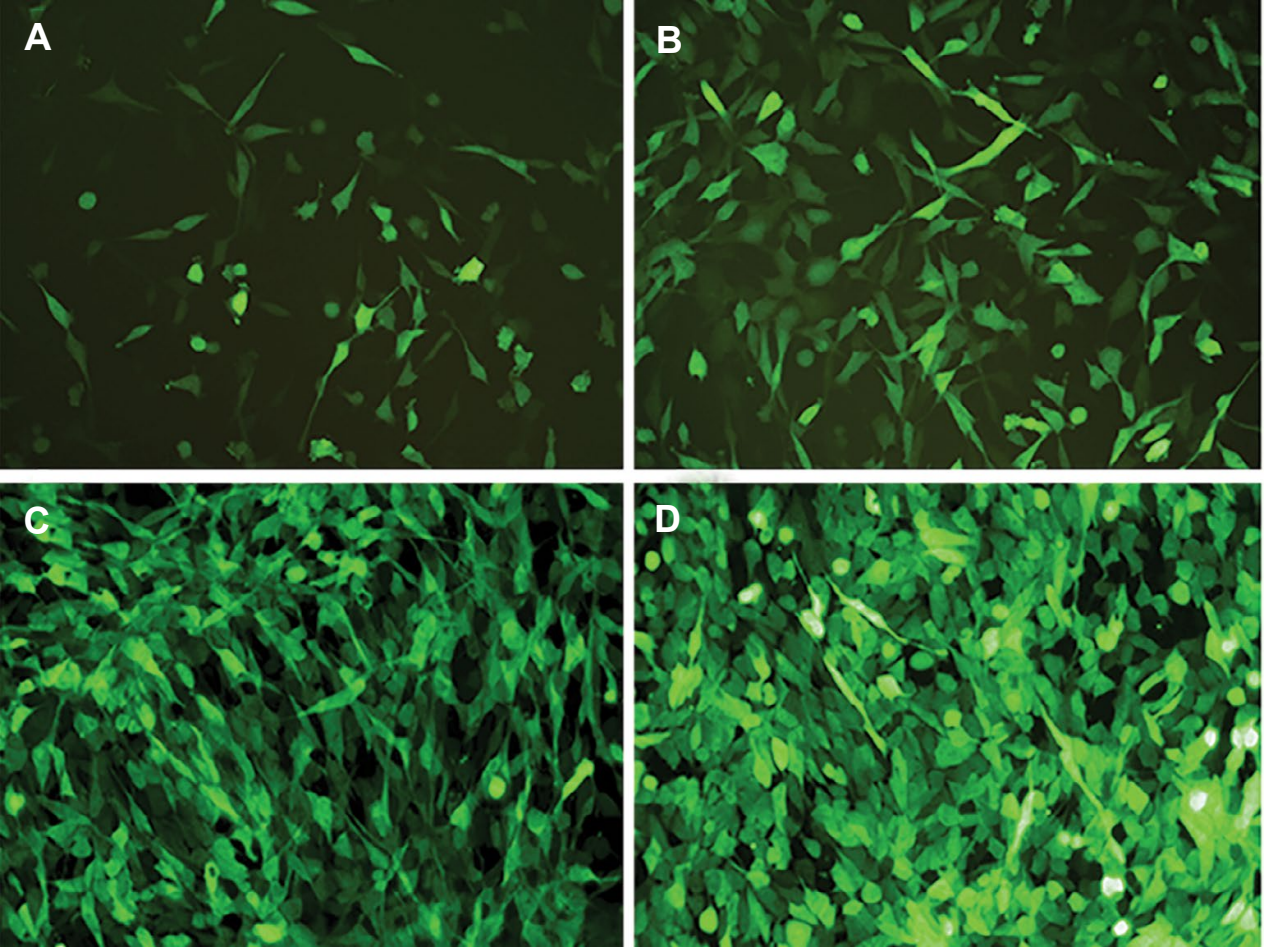
Antibiotic selection of transduced U-251 MG cell line. The stable cell line was produced by puromycin selection after 7 days. A, B. Depict the second and third day after using puromycin (2 µg/ml), C and D. Show the 5^th^ and 7^th^ day of selection.

As U-251 MG cell line highly expresses *ADK* gene, we chose it for screening anti-*ADK* miR-shRNAs (Fig .2). Nine recombinant pseudo lentiviruses (eight anti-*ADK* miR-shRNAs and scrambled control) were separately used for transduction of U-251 MG cell line. GFP reporter gene showed high efficiency of transduction and expression of anti-*ADK* miRshRNAs and scrambled pseudo lentiviruses in U-251 MG cells (Fig .3). Selection of GFP^+^ cells, 72 hours after transduction, was done by using puromycin (2 µg/ml) (Fig .4). 

### Screening of anti-*ADK* miR-shRNAs in U-251 MG cells by semi-quantitative real-time polymerase chain reaction 

**Fig.5:**
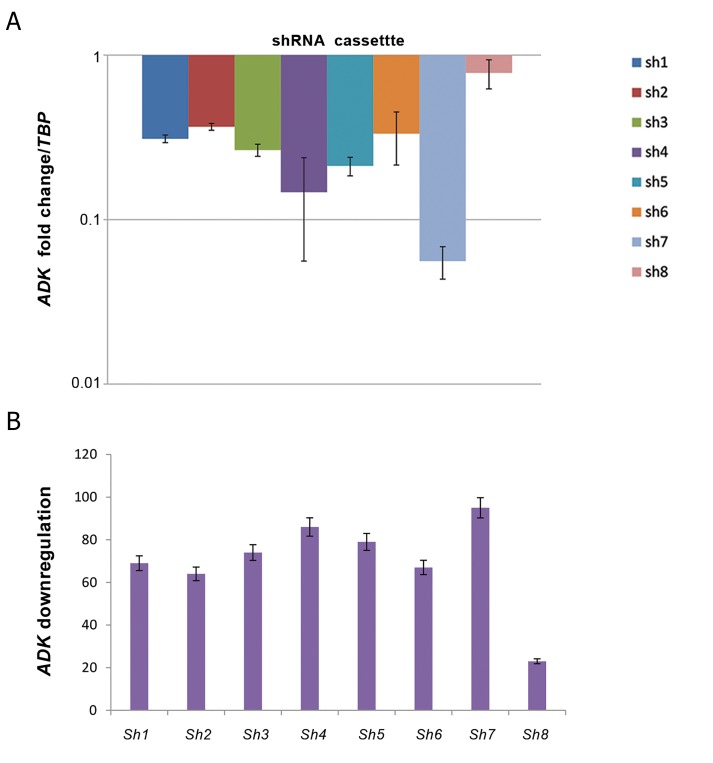
Screening of anti-*ADK* miR-shRNAs in U-251 MG cells by semiquantitative real-time polymerase chain reaction. A. The expression of ADK mRNA was evaluated in the lentiviral-engineered U-251 MG cells by REST 2009 software. For improving the reliability of real-time polymerase chain reaction (RT-PCR), *TBP* was used as a reference gene and B. Percent of *ADK* knockdown obtained by anti-*ADK* miR-shRNAs. Data showed that anti-*ADK* miR-shRNAs (except for sh8) could knock down *ADK* more than 60% and sh4 and sh7 were the most efficient with 86 and 95% *ADK* knockdown, respectively.

For selection of the most efficient anti-*ADK* miRshRNAs for *ADK* knockdown, quantitative real-time PCR was employed. Expression of *ADK* gene in each of target groups (U-251 MG cell line transduced with 8 anti-*ADK* miR-shRNAs) was measured in proportion to control group (U-251 MG cell line transduced with scrambled control). For improving the reliability of relative RT-PCR, *TBP* was used as a reference gene.Analysis by quantitative real-time PCR was done by REST2009 software (Fig .5A). Data showed that anti-*ADK* miR-shRNAs, except for sh8, could knockdown *ADK* more than 60% and sh4 and sh7 were the most efficient anti-*ADK* miR-shRNAs with 86 and 95% knockdown of *ADK*, respectively (Fig .5B).

### Morphological characteristics and mesenchymal stem cell markers expression of human Wharton’s jelly cells

**Fig.6:**
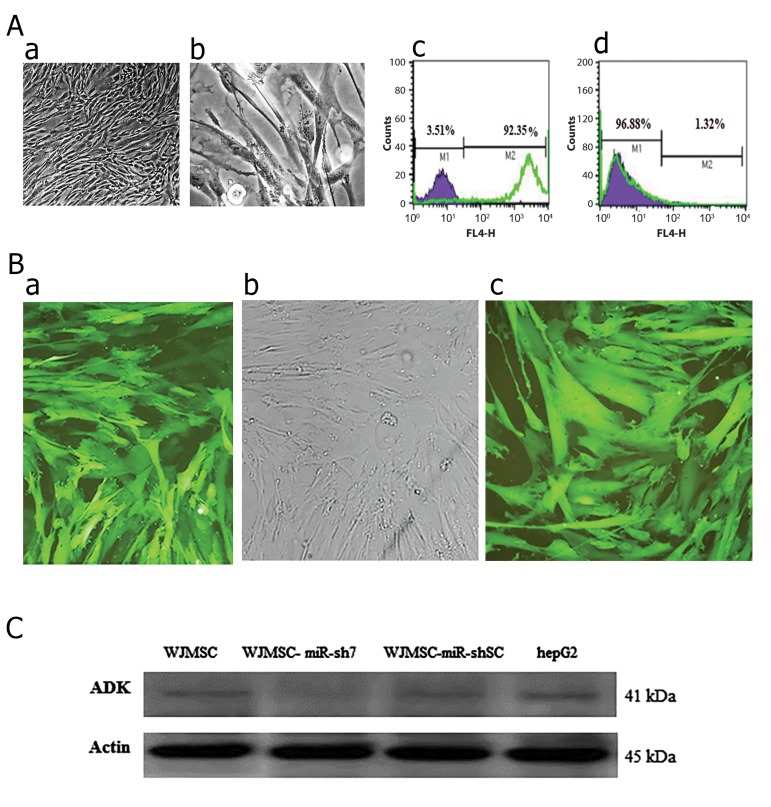
Knock down of *ADK* in human Wharton’s jelly cells. A. Morphological characteristics and mesenchymal stem cell markers expression of human Wharton’s jelly cells. WJMSCs showed fibroblast-like phenotype under a phase-contrast microscope (a, b). Flow cytometric analyses indicated that the cultured WJMSCs significantly express mesenchymal stem cell marker CD44 (c). These cells were almost negative for hematopoietic marker CD45 (d), **B.** Genetic engineering of WJMSCs by anti-*ADK* miR-sh7. WJMSCs observed under phase-contrast microscope (b). Stable transduced WJMSCs with anti- *ADK* miR-sh7 under fluorescent microscope (a). Puromycinselected WJMSCs transduced with anti-*ADK* miR-shSC (1.5 µg/ml) (c), and C. Western blot analysis confirmed *ADK* knockdown in WJMSCs. Western blot analysis was performed on cell lysates from WJMSCs, WJMSCmiR-sh7, WJMSC-miR-shSC, and HepG2. *ADK* staining (top) and ß-actin staining (bottom). WJMSCmiR-sh7 showed the most marked reduction of *ADK* expression at protein level.

Umbilical cord matrix tissue was cultured by an explant method. Afew days later, WJMSCs migrated away from tissues. After two weeks, fibroblast-like cells appeared in culture dishes. The cells grew fast and rapidly covered the surface. Flow cytometric analyses indicated that the cultured WJMSCs do not express hematopoietic marker CD45; however, they expressed mesenchymal stem cell marker CD44 (Fig .6A).

### Genetic engineering of WJMSCs by anti-*ADK* miR-sh7

Analysis of real-time data showed that Anti-*ADK* miRsh7 is the most efficient (up 95%) anti-*ADK* miR-shRNA for knockdown of this gene. For this reason, we used pseudo lentiviruses of this miR-shRNA for transduction of WJMSCs. The high efficiency of transduction was seen under the fluorescent microscope. After selection with puromycin (1.5 µg/ml) in the culture medium (Fig .6B), semi-quantitative real-time PCR was employed for measuring *ADK* expression at mRNA level. Data also showed knockdown of *ADK* gene in WJMSCs up to 95% as well as down-regulation in the U-251 MG cell line.

### Confirm of *ADK* knockdown in WJMSCs by western blot analysis 

After selection of transduced WJMSCs using anti- *ADK* miR-sh7 lentiviral vector, cell lysates were used for performing western blot. Cell lysates from transduced WJMSCs with scrambled control viruses, cell lysates from WJMSCs and HepG2 as a control were also employed. To quantify and normalizing of *ADK* immunoreactivity, ß-actin antibody were used. Analysis of data did not show any reduction of *ADK* in WJMSCs, WJMSCs-miR-shSC, and HepG2; however, down-regulation of *ADK* was observed after transducing cells with the human specific anti-*ADK* miR-sh7 (Fig .6C).

## Discussion

In adult brain, *ADK* is a key enzyme in astrocytes that regulates adenosine level by converting adenosine to 5'-adenosine monophosphate (AMP) and subsequently generates ATP (17). When trauma or epilepsy happens, adenosine, as a modulator of inflammation increases and induces proliferation of astrocytes via A2ARs, leading to astrocyte activation and *ADK* overexpression. *ADK* expression results in adenosine decline. In an acute injury or intractable epilepsy, proliferation of astrocytes (i.e. astrogliosis), is the pathological hallmark of the disease and at the molecular level, adenosine deficiency is the consequence of *ADK* overexpression induced by over-activation of astrocytes (18). So, *ADK* down-regulation and increasing adenosine release are among the strategies used for the treatment of some neurological disorders like epilepsy. Although inhibition of *ADK* with chemical and small molecule drugs can suppress seizures but systemic long term use of this therapeutic approach may increase the risk of side effects like brain hemorrhage (19). Post transcriptional gene silencing is a molecular tool that has opened new windows in recent years. Knockdown of *ADK* by viral vectors that express anti-*ADK* miRNAs can increase adenosine and results in suppressing seizures via a paracrine effect (20). Epilepsy is a chronic disease so permanent release of adenosine is needed. In this therapeutic approach, the combination of cell therapy with gene therapy is the key to the riddle. 

Cell therapy provides long-lasting focal delivery of antiepileptic molecules like adenosine and prevents systemic pharmacological side effects. Cell therapy also has the potential to restore cells that are destroyed by neurological conditions, especially in refractory epilepsy. In cell therapy approaches, safer and more controllable gene delivery of inhibitory neurotransmitters or other therapeutic compounds is possible. Chemical mutations for engineering cells to release therapeutic agents like adenosine, were induced in 2001. In that study, fibroblasts that release adenosine were generated by induction a deficiency in *ADK* and adenosine deaminase (ADA) gene (21). Transplantation of these cells implicated nearly complete protection against seizures up to 24 days but lost their antiepileptic effects after that as the viability of grafting fibroblast cells decreased. Stem cells are more attractive sources to be used for solving this problem. Stem cells in addition to having higher viability, can be differentiated into multi-lineage cells such as neuroprogenitor cells to integrate into damaged neural networks that are created during epilepsy or other neurological disorders, they can slow seizure development and they have anti-epileptogenesis effects by releasing inhibitory substances. 

Different sources of stem cells (e.g. adult stem cells, fetal stem cells, embryonic stem cells and iPS cells) have been used to evaluate treatment of animal model of epilepsy (22-25). Therapeutic application of *ADK* gene knockout in mouse embryonic stem cells and *ADK* gene knockdown in bone marrow mesenchymal stem cells have been investigated by Li et al. (26). These studies implicated reduction of seizures in a mouse model after transplantation of hMSCs and the results were promising to be used for treatment of epilepsy. However, ethical concerns about embryonic stem cells and technical issues related to bone marrow mesenchymal stem cells, have made WJMSCs as an attractive source for cell therapy and gene delivery systems.

WJMSCs could be obtained from the human umbilical cord that is normally discarded after the birth. These cells are actually a by-product of childbirth. WJMSCs have the potential to differentiate into ectodermal and mesodermal derived cells (27, 28). In addition to the easy access to WJMSCs and their potential of differentiation, these cells have anti-inflammatory and immunomodulatory properties. Some studies have shown that xenograft transplantation of WJMSCs are not rejected in an animal model of human disease without immune-suppression (29). Studies suggested that production of cytokines and growth factors by WJMSCs, results in their anti- inflammatory and neuro-protective activities (30). In the present study, we improved therapeutic benefit of WJMSCs by knocking down *ADK* gene and producing adenosine releasing WJMSCs to suppress seizures in families with status epilepticus and we stored these cells for possible future applications. WJMSCs are an attractive alternative source for cell-based gene therapy as they are i. Easily accessible stem cell sources without ethical and technical concerns, ii. Accessible in unlimited numbers and, iii. Amenable to genetic modification, and iv. They possess therapeutic potential. Based on these considerations, we selected these cells for engineering with lentiviral anti-*ADK* miR-shRNA expressing systems. 

miRNA-mediated downregulation of *ADK* was previously reported by Li et al. (26). They used five anti-*ADK* miRNA expression cassettes and showed downregulation (up to 85%) of *ADK* in human MSCs (hMSCs). In the present study, we considered human miR 30 for designing stem-loop and flanking sequences of anti-*ADK* miR-shRNAs to increase the efficiency of RNAi-based gene therapy. We used eight anti-*ADK* miRshRNAs that target *ADK* at different sense and antisense sequences but at common stem-loop and flanking sequences. 

By developing this system, we succeeded to diminish *ADK* expression up to 95% in astrocytoma cell line and in WJMSCs. ADK mRNA has 4 variants (isoform a, b, c and d). These eight anti-*ADK* miR-shRNAs can target all transcript variants of *ADK*. Antisense of sh6 and sh8 target ADK mRNA at 3´ outside of coding sequence (CDs) while other shRNAs (sh1, sh2, sh3, sh4, sh5 and sh7) target ADK mRNA at 3´ including CDs. Using human miR30 for designing shRNAs caused 60-95% knockdown of *ADK* (except for sh8 which resulted in 23% downregulation). Anti-ADK miR-sh4 and Anti- *ADK* miR-sh7 were the most efficient cassettes with up to 86 and 95% downregulation of *ADK*, respectively. These results show that the usage of miR-based shRNAs is an efficient method in knocking down *ADK*. Based on these results, in our future research, we work on using engineered WJMSC for the survey of the therapeutic potential of *ADK* down-regulation and adenosine delivery in an animal model.

## Conclusion

Results show that lentiviral system expressing anti- *ADK* miR-shRNAs that was used in this study is a promising tool for *ADK* knockdown in all transcript variants. Manipulated WJMSCs by Adeno, AAV, non- integrated lentiviral, CRISPR/Cas9 and RNA transfer instead of lentivirus, might be a suitable source in cell- based and ex vivo gene therapy of epilepsy and could be evaluated for *ADK* knockdown, *in vivo*. 
